# Gliotransmission modulates baseline mechanical nociception

**DOI:** 10.1186/1744-8069-7-93

**Published:** 2011-12-02

**Authors:** Jeannine C Foley, Sally R McIver, Philip G Haydon

**Affiliations:** 1 Department of Neuroscience, Tufts University, 136 Harrison Avenue, Boston, Massachusetts 02111, USA

**Keywords:** Adenosine, Astrocyte, Gliotransmission, Pain

## Abstract

Pain is a physiological and adaptive process which occurs to protect organisms from tissue damage and extended injury. Pain sensation beyond injury, however, is a pathological process which is poorly understood. Experimental models of neuropathic pain demonstrate that reactive astrocytes contribute to reduced nociceptive thresholds. Astrocytes release "gliotransmitters" such as D-serine, glutamate, and ATP, which is extracellularly hydrolyzed to adenosine. Adenosine 1 receptor activation in the spinal cord has anti-nociceptive effects on baseline pain threshold, but the source of the endogenous ligand (adenosine) in the spinal cord is unknown. In this study we used a transgenic mouse model in which SNARE-mediated gliotransmission was selectively attenuated (called dnSNARE mice) to investigate the role of astrocytes in mediating baseline nociception and the development of neuropathic pain. Under baseline conditions, immunostaining in the dorsal horn of the spinal cord showed astrocyte-specific transgene expression in dnSNARE mice, and no difference in expression levels of the astrocyte marker GFAP and the microglia marker Iba1 relative to wild-type mice. The Von Frey filament test was used to probe sensitivity to baseline mechanical pain thresholds and allodynia following the spared nerve injury model of neuropathic pain. DnSNARE mice exhibit a reduced nociceptive threshold in response to mechanical stimulation compared to wild-type mice under baseline conditions, but nociceptive thresholds following spared nerve injury were similar between dnSNARE and wild-types. This study is the first to provide evidence that gliotransmission contributes to basal mechanical nociception.

## Findings

Pain sensation is an adaptive response to impending tissue damage that protects an organism from extended injury. Pain perception involves a series of cellular interactions and responses from immune cells, glia and neurons. Signals from glial cells trigger neuronal responses, and vice versa, initiating a complex cascade of cell-cell interactions and feedback mechanisms [1]. A major obstacle in elucidating the relative contribution of specific cell types is reflected by the ubiquitous nature of the signaling molecules that have been implicated in the behavioral expression of pain. ATP, adenosine and glutamate are not only ubiquitous regulators of normal nervous system function, being released by and activating receptors on multiple cell types, these transmitters are also associated with many neuropathological conditions, including chronic pain.

Acute pain stimuli excite primary nociceptive neurons, which synapse and release glutamate and substance-P onto postsynaptic neurons in the dorsal horn of the spinal cord. Under chronic pain conditions, this synapse exhibits an LTP-like state where increased responses from dorsal horn neurons are elicited by afferent stimulation [2]. This is correlated with behavioral outputs such as reduced threshold to pain (allodynia) and increased severity of pain sensation (hyperalgesia). The development of chronic pain is a pathological process that is not fully understood, however experimental models of neuropathic pain (NPP) demonstrate that reactive responses of astrocytes contribute to reduced nociceptive thresholds [3], a hallmark feature of this condition, but the mechanisms by which this occurs are incompletely defined. Models of NPP also show that microglia and astrocytes residing in the dorsal horn of the spinal cord change from a resting state to an activated state, characterized by hypertrophic morphology, up-regulation of cell-specific proteins, and proliferation [4]. Astrocytes release chemical transmitters, "gliotransmitters" such as D-serine, glutamate, and ATP [5]. ATP release by astrocytes or other cell types may influence microglia [6] or neuronal responses [7] in the spinal cord. In turn, neuronal release of glutamate and substance P, both of which increase in pain, may stimulate astrocyte release of ATP [8]. Recent studies demonstrate that astrocytes also regulate adenosine 1 receptor (A1R) signaling and neuronal NMDA receptor (NMDAR) expression and synaptic plasticity [9]. Given that activation of A1Rs is anti-nociceptive [10], and since changes in NMDAR expression and activation contributes to the maladaptive synaptic plasticity associated with NPP [11], it is possible astrocytes contribute to the cellular basis of pain perception. It has been well-established that astrocyte gliotransmission represents a mechanism for fine-tuning synaptic activity [12-14], but the impact of this process as it relates to physiological behaviors, such as pain sensation, are not well-understood. The development of molecular genetic techniques in which astrocyte function is selectively impaired has made it possible to probe the behavioral impact of these glial cells. For example, using transgenic mice in which SNARE-mediated release of gliotransmitters is selectively attenuated (called "dnSNARE" mice), astrocytes were shown to be critical regulators of A1R-dependent sleep homeostasis [15]. In this study, dnSNARE mice were used to probe the contribution of gliotransmission to physiological and pathological pain sensation.

**Figure 1:**
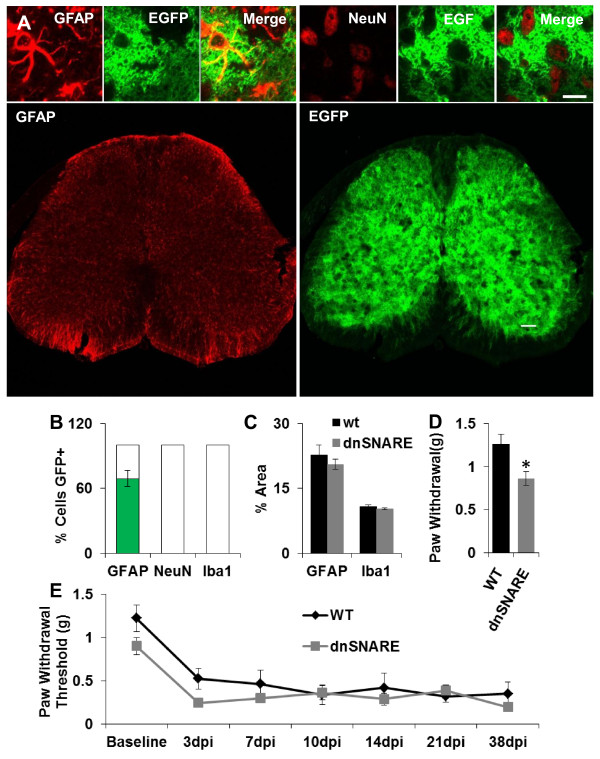
**Astrocyte Specific Attenuation of Gliotransmission Causes Reduced Basal Nociception but Does Not Alter NPP**. (A) The lumbar spinal cord exhibits abundant expression of EGFP reporter protein (green) with distinct colocalization with the astrocyte marker, GFAP (red; left), but not with the neuronal marker NeuN (red; right). (B) Quantification of EGFP+ cells reveals that 69.2 ± 7.3% GFAP+ cells were EGFP+ in dnSNARE mice but 0% colocalization was present Iba1+ or NeuN+ cells. No EGFP+ cells were found in WT sections (not shown). (C) dnSNARE expression does not cause reactive astrocytes or microglia as shown by similar GFAP (WT: 22.7 ± 2.4% n = 3; dnSNARE: 20.6 ± 1.2% n = 3; P = 0.24) and Iba1 (WT: 10.7 ± 0.5% n = 3; dnSNARE: 10.3 ± 0.2% n = 3; P = 0.20) staining between dnSNARE and WT dorsal horns. (D) DnSNARE mice exhibit a significant reduction in baseline paw withdrawal thresholds compared to WT mice (WT: 1.24 ± 0.13 n = 18, dnSNARE: 0.86 ± 0.08 n = 21 *P < 0.01). (E) DnSNARE and WT mice both exhibit sustained reduction in paw withdrawal threshold after SNI with no significant difference between WT and dnSNARE (WT: n = 9 for 3-21 dpi, n = 7 for 28 dpi dnSNARE: n = 12 for 3-21 dpi, n = 7 for 28 dpi P = 0.570). Scale bars: 10 μm (upper); 100 μm (lower). dpi, days post injury.

DnSNARE mice were created by crossing two lines of transgenic mice using the tetracycline regulatory system: in one line of mice, the astrocyte-specific Glial Fibrillary Acidic Protein (GFAP) promoter was used to drive expression of tetracycline transactivator, and in the other line, the tetracycline operator was used to drive expression of the enhanced green fluorescent protein (EGFP) reporter gene and dominant-negative expression of the cytosolic portion of the synaptobrevin complex (dnSNARE) to attenuate vesicle fusion [12]. This system allows for inducible transgene expression through removal of doxycycline from the diet. Previous studies show astrocyte-selective transgene expression throughout the brain, including the cortex, hippocampus, and basal forebrain [12,15]. To confirm astrocyte-specific transgene expression in the spinal cord, levels L4 to L6 were histologically examined. Immunostaining against GFAP shows colocalization with the EGFP transgene, whereas the microglial marker Iba1 and the neuronal marker NeuN do not (n = 3; EGFP+/GFAP+ cells = 69.2 ± 7.3%; EGFP+/NeuN+ and EGFP+/Iba1+ cells = 0; P < 0.001) suggesting that dnSNARE is expressed selectively in astrocytes (Figure 1A, B). Reactive responses in astrocytes and microglia, quantified as percent area of substantia gelatinosa above background staining of GFAP (WT: 22.7 ± 2.4% n = 3; dnSNARE: 20.6 ± 1.2% n = 3; P = 0.24) and Iba1 (WT: 10.7 ± 0.5% n = 3; dnSNARE: 10.3 ± 0.2% n = 3; P = 0.20), are similar in the spinal cords of dnSNARE and WT mice (Figure 1C).

Since astrocytic expression of dnSNARE causes reduced A1R activation [15], and stimulation of this receptor in the spinal cord has anti-nociceptive effects on baseline pain [10], we hypothesized that dnSNARE mice would have altered basal nociception. We measured mechanical nociception in WT and dnSNARE adult male mice, aged 8-10 weeks, using von Frey filaments, a method in which a constant pressure is applied to the plantar surface of the hind paw and subsequent paw withdrawal threshold is measured. DnSNARE mice exhibit a reduced threshold to mechanical nociception (WT: 1.24 ± 0.13 n = 18; dnSNARE: 0.86 ± 0.08 n = 21; p < 0.01), suggesting they have increased baseline sensitivity to pain (Figure 1D). Given that acute pain sensation is a spinal reflex, we anticipate that this effect is mediated by dorsal horn astrocytes. However, we cannot rule out the potential contribution of supraspinal brain regions, such as the rostral ventral medulla or anterior cingulate cortex, where dnSNARE expressing astrocytes are also present (not shown). Furthermore, it is unlikely that satellite glial cells in the dorsal root ganglion are involved as GFAP is not highly expressed in these cells before injury [16].

Given that baseline nociception is altered in dnSNARE mice, and since these mice are known to exhibit reduced release of ATP, a purine known to play important roles in neuropathic pain, we asked whether astrocytic dnSNARE expression alters the development of NPP. The spared nerve injury (SNI) method of NPP [17-19] was used to test this hypothesis. One day following measurement of baseline nociception, mice were anesthetized and the tibial and peroneal branches of the sciatic nerve were ligated, sparing the sural branch. Animals were allowed to recover from surgery and Von Frey monofilaments were used to test NPP at 3, 7, 10, 14, 21, and 28 days post-injury (dpi). Despite a clear difference in basal pain perception, dnSNARE mice exhibit progressive development of NPP after SNI, similar to WT mice (FIG 1D; WT: n = 9 for 3-21 dpi, n = 7 for 28 dpi; dnSNARE: n = 12 for 3-21 dpi, n = 7 for 28 dpi; P = 0.570).

This study shows that gliotransmission modulates physiological mechanical nociception. Given that administration of A1R agonists are known to decrease pain responses [10] and that previous studies show dnSNARE mice exhibit reduced adenosine tone [15], we speculate that the reduced baseline pain threshold measured in dnSNARE mice is due to decreased A1R activation in the spinal cord. This would suggest that an astrocytic source of adenosine contributes to acute pain signaling in the spinal cord. However, we cannot discount the possibility of additional contributions from other gliotransmitters such as D-serine [20].

Astrocytes have been recently promoted from being merely the "glue" that supports neurons, to key players in synaptic activity and homeostatic regulators of nervous system function. We now know they are integrally involved in neuronal activity and are starting to learn more about how astrocyte function impacts behavior. Our results showing that a mouse model of attenuated gliotransmission exhibits a reduction in baseline mechanical nociception is the first indication that astrocytes modulate physiological pain behavior.

## Methods

### dnSNARE mouse

The tTA tet-O system was used to create inducible astrocyte-specific expression of dnSNARE, a dominant negative mutation of the synaptobrevin II protein in the SNARE complex. Two mouse lines were crossed to achieve inducible expression of dnSNARE selectively in astrocytes, the tetracycline transactivator line was driven by the astrocyte specific GFAP promoter, and the dnSNARE, GFP and Lac-Z reporter genes were driven by the tetracycline operator [12,15,21]. Mice were raised on doxycycline to prevent transgene expression during development and thus to prevent potential adaptations to transgene expression. Doxycycline was removed from the diet at weaning to permit transgene expression in the mature animal. Two weeks post doxycycline removal, abundant transgene expression was evident throughout the brain and spinal cord. Male animals were tested at 8-10 weeks of age and were housed on a 12 hr/12 hr light/dark cycle. All procedures were in strict accordance with National Institutes of Health Guide for Care and Use of Laboratory Animals and were approved by the Tufts University Institutional Animal Care and Use Committee.

### Behavioral Testing

Von Frey monofilaments were used to test baseline mechanical nociception as well as allodynia after SNI. The monofilaments were used to apply controlled force to the lateral portion of the left hind limb, similar to what has been previously described [17-19]. Animals were tested in wire mesh chambers to which they were habituated 5 days before testing. Pain threshold was recorded when animals elicited 4-5/10 pain responses (defined by rapid paw withdrawals often with paw shaking or licking) for a given monofilament ranging from 0.02 g to 2 g. A subset of animals was tested for NPP after SNI. Surgery was performed under isoflurane anesthesia and the tibial and peroneal branches of the sciatic nerve were ligated while the sural branch was spared [17]. Beginning 3 dpi, changes in mechanical nociceptive responses were measured using von Frey's fiber test. Measurements were taken as described above, and trials were repeated on 3, 7, 10, 14, 21, and 28 dpi. Criteria for exclusion identified outliers (> 2 standard deviations from within group mean), which were excluded from this study (2 dnSNARE, 1 WT). Upon completion of the experiments the animals were euthanized by isoflurane followed by cardiac perfusion, for histology.

### Immunohistochemistry

Mice (3 wild-type and 3 dnSNARE) were transcardially perfused with 4% paraformaldehyde in phosphate buffered saline (PBS) and post-fixed for 24 hours. Spinal cords were removed from spinal column and placed in 10% and then 30% sucrose. The lumbar spinal cord (L4-L6) was sectioned on a sliding microtome at a thickness of 40 μm and placed in PBS. Sections were stained with Rabbit anti-Iba-1 (Wako, 1:1000) chicken anti-GFAP (abcam, 1:1000), and mouse anti-Neu-N (chemicon, 1:1000). Secondary antibodies conjugated to Alexafluor were used. Goat anti-rabbit Alexa633, goat anti-chicken Alexa546 and goat anti-mouse Alexa633 were used, all at a 1:500 dilution. At least three representative sections were taken from each condition for quantification.

### Confocal imaging

Confocal images were acquired using a Nikon Eclipse Ti microscope. The substantia gelatinosa of the dorsal horn was imaged. For imaging of reactive changes in astrocytes and microglia, maximum intensity projections were created from 10 μm Z-stacks taken with a 20× objective (0.75 NA). ImageJ software was used to measure the percent area above threshold for each antibody. Background values were subtracted. For counting of co-localization, single plane optical section images (60× objective, 1.40 NA) were taken and each clearly defined cell was counted as EGFP positive or negative based on localization of astrocyte, microglia or neuron markers with DAPI labeled nuclei.

### Statistics

Student's t-test was used to determine significance between baseline pain groups and IHC quantification. Repeated Measures 2-Way ANOVA was used to determine whether a difference existed in NPP development between genotypes. Raw data were transformed to Log10 for ANOVA analysis.

## List of abbreviations used

NPP: neuropathic pain; A1R: adenosine 1 receptor; SNI: spared nerve injury; GFAP: glial fibrillary acidic protein; dpi: days post injury; EGFP: enhanced green fluorescent protein; NMDAR: N-methyl D-aspartate Receptor.

## Competing interests

PH has equity interest in Gliacure Inc.

## Authors' contributions

JF and SM contributed equally to the manuscript. JF, SM, and PH designed the experiments. Experiments and data analysis were carried out by JF and SM. JF, SM drafted the manuscript. All authors read and approved the final manuscript.
